# Epidemiology and Performance After Achilles Tendon Rupture in National Basketball Association (NBA) Players: A Retrospective Case Series Rationale

**DOI:** 10.7759/cureus.114162

**Published:** 2026-08-08

**Authors:** Owen Kennedy, Owen Hallauer, Sean H Gordon, Abraham Hussain, Scarlett Schneider

**Affiliations:** 1 Orthopaedic Surgery, Medical College of Georgia, Augusta University, Augusta, USA; 2 Vascular Surgery, Mercer University School of Medicine, Savannah, USA; 3 Sports Medicine, Augusta University/University of Georgia Medical Partnership, Athens, USA

**Keywords:** achilles, achilles tendon rupture, basketball, calf strain, nba

## Abstract

Background

Achilles tendon (AT) ruptures are among the most severe injuries sustained by National Basketball Association (NBA) players, with prolonged recovery times and documented long-term impact on performance. The 2024-2025 season witnessed a record-high number of these injuries, including multiple high-profile cases, prompting renewed interest in understanding the burden and epidemiological trends of AT ruptures in professional basketball.

Purpose

The purpose of this study is to characterize the epidemiology of complete AT ruptures in NBA players over the past 15 seasons and explore trends in player demographics and performance after return to play.

Methods

A retrospective review was conducted using publicly available data on NBA players who experienced complete AT ruptures between 2010 and 2025. Inclusion was limited to players who returned to NBA competition within two seasons and had no prior same-side lower-extremity (LE) surgeries. Pre- and post-injury minutes per game (MPG) and player efficiency rating (PER) were compared using paired t-tests. Subgroup analyses were performed based on age, height, and weight.

Results

Among the 12 included players, the average age at the time of injury was 27.9 years, with an average of 9.25 months missed. There was a significant decline in MPG post-injury (p = 0.0009, d = 1.31), while the decline in PER trended toward significance (p = 0.0072, d = 0.95). On average, MPG and PER decreased by 20.2% and 21.9%, respectively. Younger players (≤28 years) represented a growing share of recent injuries.

Conclusion

AT ruptures result in substantial decreases in performance and prolonged absences, with possible trends toward earlier injury onset. Further research is needed to elucidate the causes of this recent increase in such a profound injury.

## Introduction

The incidence of Achilles tendon (AT) ruptures has increased over the years [1,2]. Recently, three high-profile cases in the 2025 National Basketball Association (NBA) playoffs once again brought AT ruptures to the forefront, capping off a season that saw a record-high seven of these injuries [3]. Comparatively, there were zero AT ruptures in the previous season [4]. While other lower-extremity (LE) injuries, such as lateral ankle sprains, patellofemoral inflammation, and hamstring strains, are some of the most common injuries faced by NBA players [5], AT ruptures are among the most catastrophic injuries in professional basketball, with an average recovery time of 10.5 to 11 months in NBA athletes [6,7].

Basketball players are particularly susceptible to AT ruptures due to the high mechanical load experienced by the ankle joint and the physical demands they encounter throughout their careers [7,8]. The minimum timeline for return to play (defined as first appearance following injury) after an AT rupture is typically six months, and the quality of play following an AT rupture is decreased compared with before the injury [8]. A subset of athletes may even miss two years or fail to return to play altogether [7].

Of the 70.8% of players who return to play within two seasons following AT repair, many often face hindered performance and shortened careers [9,10]. Player efficiency rating (PER) is a relatively new measure that takes into account several statistics to grade a player’s overall performance [11], and PER in NBA players who return to play after an AT rupture is significantly decreased [9]. Other measures can be used to track NBA player performance following an injury, such as minutes per game (MPG), value added, and estimated wins added metrics [12].

The purpose of this report is to highlight the epidemiological trends and burdens associated with AT rupture in the NBA over the past 15 seasons. Using this data, we aim to formulate a series of hypotheses to explain the recent increase in these injuries and better characterize the impact on players after their injury.

## Materials and methods

We conducted a retrospective review of AT ruptures in NBA players from 2010 through 2025. Cases were identified from publicly available sources, including NBA.com Injury Report Archive, ESPN Injury Tracker, Basketball Reference, and validated news reports (see supplemental sheet, Appendix 1) [13-15]. For each player, multiple reports from the day the injury occurred were cross-checked to ensure accuracy and confirm the correct date. The inclusion criteria for this review consisted of NBA players who sustained an AT rupture between 2010 and 2025 and subsequently returned to NBA competition within two seasons. Exclusion criteria included players who had a history of same-side LE surgery prior to their AT rupture to better isolate the effects of AT rupture on player performance [16].

The data collected included age at injury, height, weight, dates of injury and return to play, pre-injury and post-injury MPG, pre-injury and post-injury PER, and months missed due to injury. MPG and PER were based on the season of the injury, unless the player was injured before five games, in which case the previous season's MPG average was used. Post-injury MPG and PER were taken from the next season following injury. These variables were selected instead of points per game since not all players add their value to the team through scoring. PER and MPG provide a more holistic evaluation of a player's value to the team and do not discriminate against primarily defensive players.

The primary analysis of this data utilized paired t-tests to determine if there was a statistically significant difference between pre-injury and post-injury MPG and PER. MPG assesses how much a player was utilized by their team, and PER summarizes their statistical contributions into a single per-minute number, which standardizes a player's contributions and efficiency. A significant difference would support the conclusion that AT ruptures hinder a player's ability to return to play at the same level of efficiency and production, even after a full recovery. Given the small sample size, the normality of within-player differences was assessed using the Shapiro-Wilk test. Additionally, this study had several exploratory aims: descriptive trends in AT rupture frequency, time missed, and subgroup analyses by age (>28 vs. ≤28) were examined using paired t-tests. This age cutoff was based on the average age of players (27.9 ± 4.0 years) who suffered AT ruptures in our cohort. The subgroup analysis was performed to determine whether there were significant differences by age at rupture, which could highlight a potential confounder. The height and weight of players who suffered AT ruptures were compared with the NBA mean height and weight using two-sided t-tests. Microsoft Excel (Microsoft 365; Microsoft® Corp., Redmond, WA, USA) was used for all statistical analyses, with p < 0.05 considered significant.

## Results

**Figure 1:**
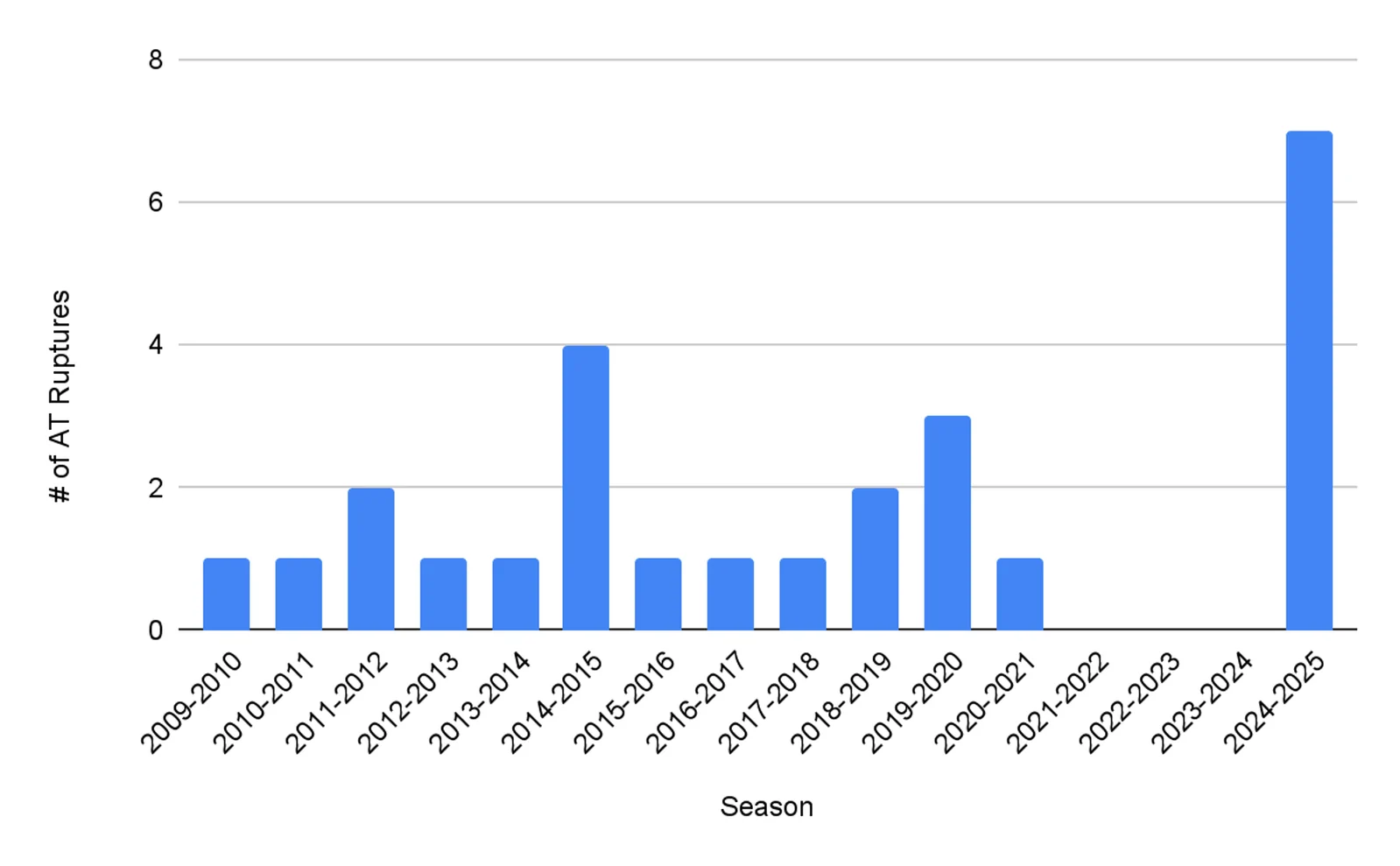
Trend of complete Achilles tendon (AT) ruptures in NBA players from the 2009-2010 season to the 2024-2025 season (26 total AT ruptures) NBA: National Basketball Association

Figure 1 describes the trend of complete AT ruptures in NBA players from the 2009-2010 season to the 2024-2025 season, highlighting the recent uptick in 2024-2025. For the primary aim of this study, paired t-tests were used to assess the statistical significance of the differences between pre-injury and post-injury MPG and PER (Table 1). We found a significant difference between pre-injury and post-injury MPG (p = 0.0009) and PER (p = 0.0072). Shapiro-Wilk testing showed no significant departure from normality for the paired differences in either MPG (p = 0.124) or PER (p = 0.245). To measure the effect size of the injury on MPG and PER, Cohen's d was calculated. Our results showed that Achilles ruptures had a significant effect on both MPG (d = 1.309) and PER (d = 0.950) in our sample population.

**Table 1:** Comparison of pre- and post-injury performance metrics in NBA players with AT (Achilles tendon) ruptures NBA: National Basketball Association

Variable	Pre-injury Mean	Post-injury Mean	Mean Difference	95% CI	p-value	t-stat	Cohen’s d value
MPG	30.9	24.5	-6.4	-9.46 to -3.28	0.0009	4.54	1.31
PER	16.8	13.3	-3.5	-5.92 to -1.18	0.0072	3.29	0.95

Across the 12 players who have returned from AT ruptures in the last 15 seasons, the average age was 27.9 years, compared with the lower average age of the 2024-2025 season ruptures, which was 26.4 years. To test for a potential impact of age at injury on MPG and PER at return to play, another paired t-test compared players <27.9 years with those >27.9 years. No statistically significant difference was observed for either MPG (p = 0.605) or PER (p = 0.231) between the two subgroups (Table 2). Other findings are illustrated in Table 3.

**Table 2:** Comparison of performance metrics in NBA players who sustained AT (Achilles tendon) ruptures before and after turning 28 years old NBA: National Basketball Association; MPG: Minutes per Game; PER: Player Efficiency Rating

Variable	<28 y/o mean	>28 y/o mean	Mean difference	p-value	t-stat	Cohen’s d value
% MPG decrease	22.7%	17.5%	5.2%	0.60	0.30	0.55
% PER decrease	29.5%	14.3%	15.2%	0.23	1.28	0.31

**Table 3:** Trends in player demographics, recovery, and return to play performance in AT (Achilles tendon) ruptures from the last 15 years after exclusion criteria were applied MPG: Minutes per Game; PER: Player Efficiency Rating

Measure	Value
Achilles ruptures included (n)	12
Average months missed (range)	9.25 (4-13)
Average % decrease in MPG (actual decrease)	20.20% (-6.37)
Average % decrease in PER (actual decrease)	21.90% (-3.55)
% Achilles tendon ruptures (ATRs) during playoffs	19.2% (5/26)
Average age of the included group	27.9
Average height (inches)	78.9
Average weight (pounds)	226.7

## Discussion

This study aims to illustrate the impact of AT ruptures on the efficiency and playing ability of NBA players over the past 15 years, which is of particular interest given the seven AT ruptures observed during the most recent NBA season. Our findings show that AT ruptures can have profound long-term implications for an NBA player's career. Despite a previous epidemiological study reporting that AT ruptures most often occur later in the season in veteran players [2], recent trends suggest that younger players are just as susceptible to injury at the end of the season or during the postseason. The average number of months a player misses after suffering an AT rupture is 9.25 months, markedly shorter than the 11-month average absence reported in a 2021 systematic review [6].

Previous data have shown that approximately one-third of NBA players who experience an AT rupture either do not return to play or start in fewer than 10 games, leading to early retirement [2]. Still, our data show that in recent history, only 1 of the 19 players who suffered an AT rupture was unable to return to play. While this is promising, an AT rupture can have significant long-term effects on a player's performance when they do eventually return to play. We found that following an AT rupture, a player's MPG decreased by an average of 6.4. Additionally, PER decreased by an average of 3.5, which is comparable to the findings reported by Khalil et al. [16]. Despite these decreases in player performance, only one player did not return to play at all. This finding is encouraging, as it reflects a substantially higher return-to-play rate than the previously reported 21% retirement rate following AT ruptures from 1970-2018 [2]. This improvement can likely be attributed to advances in surgical techniques, sports medicine, and rehabilitation programs available to players today [17]. Khalil et al. [16] also found that the average age of a player who experienced an AT rupture and returned to play was 28.22 ± 4.13 years. We found the average age of all players who experienced an AT rupture over the past 15 years to be 27.9 ± 4.0 years. In the most recent NBA season, the average age of a player who experienced an AT rupture was 26.4 years. Although these sample sizes are too small to establish statistical significance, they may suggest a trend toward NBA players sustaining AT ruptures at a younger age. Previous studies have compared the performance of players returning from AT rupture with matched peers and have shown that players who return from AT rupture have lower PER and MPG than their matched counterparts [16].

The retrospective observational design of this study limits causal conclusions; however, multiple factors could be contributing to the observed trends. NBA players today experience substantial cumulative stress on their tendons throughout their careers. Many begin playing competitively in AAU basketball and continue year-round through high school. They then spend a significant portion of the year competing in college, followed by the demands of an 82-game NBA season. Repetitive microtrauma may contribute to preexisting tendinopathy, making the AT more susceptible to rupture during an acute plantarflexion event. When players advance deep into the postseason, they may have only a few months of recovery before preparing for the following season. To remain competitive, many continue intensive training throughout the offseason, further contributing to cumulative tendon loading and repetitive microtrauma. Players are also training to become more explosive so they can move faster, change direction more quickly, and jump higher than their opponents to gain a competitive advantage. Although this training has improved the quality of play in the NBA, the greater forces generated by the players' leg musculature place increased stress on the AT, and sufficiently high loads may cause the tendon to fail, resulting in an AT rupture. 

One limitation of this study is its small sample size, given that an AT rupture is a relatively rare injury in NBA players. This may result in limited statistical power, reduced precision, increased type II error, limitations in subgroup analyses, or the inability to perform multivariable analyses. Given that players who failed to return within two seasons were excluded from the study, there is potential for survival bias and an underestimation of the true decline in performance. For injury reporting, we only had access to injuries that were sustained during each player's NBA career. A potential, but unlikely, confounding factor would be a serious, same-side LE injury that occurred earlier in a player's career. This could include injuries such as previous ankle fractures and calf strains. This study relies on publicly reported data, which increased the transparency of our analysis. However, such datasets can contain reporting inaccuracies and inconsistencies, which have the potential to introduce selection bias and reduce precision. It is difficult to attribute the decline in a player's PER and MPG solely to an AT rupture, as NBA teams frequently make roster adjustments during the offseason. These changes are influenced by a variety of factors, including injuries and the natural aging process. Another limitation is that each team has its own return-to-play protocol, and the decision for a player to return is often guided by the team doctor, athletic trainers, coaches, and the player themself. Different surgeons may employ various surgical techniques and approaches to repair the AT rupture, and rehabilitation programs may be tailored on a case-by-case basis. Lastly, this study focuses solely on complete AT ruptures, which require operative repair. Other Achilles pathologies, such as partial AT ruptures and Achilles tendinitis, may also impact a player's performance on the court and their utilization by their team. Still, these injuries may be underreported and, as such, pose difficulties in gathering meaningful data. Further studies should be conducted to determine the most effective operative timing, surgical techniques, and rehabilitation programs for maximizing a player's PER and MPG following a complete AT rupture. Lastly, it is important to note that the results of this study apply only to NBA athletes; they cannot be generalized to recreational athletes, female athletes, or athletes participating in other sports. 

## Conclusions

The recent increase in AT ruptures, particularly in star players, is concerning and warrants further investigation. Despite the lower rates of retirement and reduced missed time stemming from AT ruptures, there is still a significant decline in player performance, as well as a slightly lower average age of rupture compared with historical predictions. This study offers hypotheses on why AT ruptures are trending in this direction, including increased offseason training, higher intensity of play, and missed diagnosis of microtrauma in the tendon preceding the complete tear. In reality, it is likely a combination of factors, presenting an opportunity for future research.

## Appendices

Appendix 1

**Table 4:** Supplementary resource table

Player name	Source link
Mehmet Okur	https://www.espn.com/nba/playoffs/2010/news/story?id=5109836
Darrell Arthur	https://www.espn.com/nba/story/_/id/7384343/memphis-grizzlies-darrell-arthur-surgery-repair-torn-achilles-tendon-miss-season
Chauncey Billups	https://www.espn.com/los-angeles/nba/story/_/id/7552138/los-angeles-clippers-chauncey-billups-season-torn-achilles
Kobe Bryant	https://www.espn.com/los-angeles/nba/story/_/id/9166874/kobe-bryant-los-angeles-lakers-probably-tore-achilles-team-says
Jeff Taylor	https://www.espn.com/nba/story/_/id/10177313/jeff-taylor-charlotte-bobcats-season-achilles-injury
Brandon Jennings	https://www.espn.com/nba/story/_/id/12227137/brandon-jennings-detroit-pistons-season-ruptured-left-achilles-tendon
Wesley Matthews	https://www.espn.com/nba/story/_/id/12430843/wesley-matthews-portland-trail-blazers-injures-left-foot
Rudy Gay	https://www.espn.com/nba/story/_/id/18513578/sacramento-kings-forward-rudy-gay-fully-ruptured-achilles
DeMarcus Cousins	https://www.espn.com/nba/story/_/id/22228947/demarcus-cousins-new-orleans-pelicans-tears-achilles
Rodney Hood	https://www.espn.com/nba/story/_/id/28240968/trail-blazers-rodney-hood-tears-left-achilles-tendon
Dwight Powell	https://www.espn.com/nba/story/_/id/28534367/mavs-dwight-powell-ruptures-right-achilles-clippers
Klay Thompson	https://www.espn.com/nba/story/_/id/30349208/source-golden-state-warriors-klay-thompson-suffers-season-ending-achilles-tear
James Wiseman	https://www.espn.com/nba/story/_/id/41985010/pacers-center-james-wiseman-torn-achilles-sources-say
Isaiah Jackson	https://www.espn.com/nba/story/_/id/42150111/sources-pacers-isaiah-jackson-suffers-torn-achilles-tendon
Dru Smith	https://www.espn.com/nba/story/_/id/43142951/sources-heat-dru-smith-torn-achilles-done-season
Dejounte Murray	https://www.espn.com/nba/story/_/id/43645701/pelicans-murray-suffers-lower-right-leg-injury-vs-celtics
Damian Lillard	https://www.espn.com/nba/story/_/id/44893873/bucks-damian-lillard-exits-vs-pacers-lower-leg-injury
Jayson Tatum	https://www.espn.com/nba/story/_/id/45131551/celtics-jayson-tatum-undergoes-surgery-torn-achilles
Tyrese Haliburton	https://www.espn.com/nba/story/_/id/45564723/pacers-tyrese-haliburton-helped-lower-right-leg-injury-game-7-nba-finals-vs-thunder
Jonas Jerebko	https://www.espn.com/nba/news/story?id=5655759
JJ Barea	https://www.espn.com/nba/story/_/id/25740516/dallas-mavericks-guard-jj-barea-torn-achilles-tendon
Xavier Henry	https://www.espn.com/los-angeles/nfl/story/_/id/11931809/xavier-henry-los-angeles-lakers-suffered-left-achilles-injury
Anderson Varejao	https://www.espn.com/nba/story/_/id/12076383/anderson-varejao-cleveland-cavaliers-torn-achilles
Mario Chalmers	https://www.espn.com/nba/story/_/id/14946874/memphis-grizzlies-waive-mario-chalmers-achilles-tendon-tear
Kevin Durant	https://www.espn.com/nba/story/_/id/26944533/sources-durant-flies-new-york-undergo-mri
John Wall	https://www.espn.com/nba/story/_/id/25931300/john-wall-washington-wizards-ruptures-achilles-tendon-miss-approximately-12-months
